# The *Leptospira interrogans* CdaA protein is a functional diadenylate cyclase

**DOI:** 10.1128/iai.00716-25

**Published:** 2026-02-19

**Authors:** Edward J. A. Schuler, Dhara T. Patel, Aidan D. Moylan, Daniel P. Miller, Richard T. Marconi

**Affiliations:** 1 Department of Microbiology and Immunology, Virginia Commonwealth University Medical Center 72054, Richmond, Virginia, USA; University of California Davis, Davis, California, USA

**Keywords:** c-di-AMP, CdaA, diadenylate cyclase, *Leptospira*, leptospirosis, LIC10844, osmoregulation

## Abstract

Leptospirosis is a zoonotic disease that affects humans, companion animals, livestock, and wildlife. There are over 60 species that are established pathogens. Leptospires must rapidly adapt to changing environmental conditions as they pass between the environment and vertebrates. Bioinformatic analyses have identified a putative CdaA-type diadenylate cyclase (DAC) in *Leptospira interrogans* Fiocruz L1-130 (*lic10844*). DACs catalyze the synthesis of cyclic di-adenosine monophosphate (c-di-AMP) from two ATP molecules. The potential regulatory roles and effector mechanisms of c-di-AMP among pathogenic *Leptospira* species have not been explored. Here, we demonstrate that *lic10844* encodes a functional DAC (henceforth referred to as CdaA). Cellular localization analyses, size exclusion chromatography, and DAC assays revealed that CdaA is an inner membrane-associated protein that functions biologically as a homodimer, utilizing cobalt or manganese for enzymatic activity. Transcription of *cdaA* is responsive to and elevated by potassium levels. Individual amino acid residues that directly or indirectly mediate the DAC activity of CdaA were identified using site-directed mutagenesis. This report represents an important initial step in elucidating the biological function of CdaA, and by extension, c-di-AMP, in the biology and pathogenesis of *Leptospira* species.

## INTRODUCTION

Leptospirosis, the most common zoonotic disease worldwide, poses a significant health threat to humans, companion animals, livestock, and wildlife (1). The causative agents are a group of genetically and antigenically diverse *Leptospira* species. There are approximately 1 million human cases of leptospirosis each year, resulting in nearly 60,000 deaths (2, 3). However, due to limited diagnostic tools and reporting infrastructure, the actual number of cases is likely much higher (2). Pathogenic *Leptospira* species penetrate mucosal membranes and skin abrasions and subsequently establish an infection in the renal tubules. Leptospires are shed back into the environment through urine.

The transmission cycle of *Leptospira* species mandates the need to rapidly adapt to radically different environments (diverse vertebrate hosts and free-living). Bioinformatic analyses have identified an extensive repertoire of putative regulatory proteins involved in environmental sensing and adaptation (1). *Leptospira* spp. harbor several genes that encode diguanylate cyclases (DGC) and a single gene encoding a diadenylate cyclase (DAC). DGCs and DACs catalyze the production of the secondary messenger molecules, cyclic-di-GMP (c-di-GMP) and cyclic-di-AMP (c-di-AMP), respectively. The c-di-GMP regulatory systems of spirochetes, including *Borreliella burgdorferi* (4–13), *Borrelia hermsii* and *B. turicatae* (12, 14), *Treponema denticola* (4, 15), *L. interrogans* (16–18), and *L. biflexa* (19), have been studied to varying degrees. Gene deletion of the sole DGC (*rrp1*: response regulatory protein 1) of the Lyme disease spirochete, *B. burgdorferi*, revealed that Rrp1, and by extension, c-di-GMP, regulates over 10% of the transcriptome and is essential for spirochete survival within ticks (8, 20). C-di-GMP levels have been shown to regulate biofilm formation in *L. interrogans* and to contribute to environmental stress responses (16).

The protein network required for the synthesis and degradation of c-di-AMP has been predominantly identified and characterized in gram-positive bacteria (21). C-di-AMP has been demonstrated to contribute to osmotic homeostasis, peptidoglycan biosynthesis, and stress responses. The regulatory role and effector mechanisms of c-di-AMP in spirochetes are less understood. Moylan et al. demonstrated that the periodontal pathobiont, *T. denticola*, encodes a single CdaA-type DAC (19), demonstrating *de novo* c-di-AMP synthesis *in vitro*. The Lyme disease (*B. burgdorferi*) and tick-borne relapsing fever (*B. turicatae*) spirochetes also encode a CdaA-type DAC (22–24). Suppressor mutations of *cdaA* in *B. turicatae* resulted in attenuated virulence in mice, revealing the necessity of c-di-AMP for normal growth, particularly in the presence of salts.

Given the complex environmental conditions that *Leptospira* spp. encounter in vertebrates and host-free environments, c-di-AMP is likely a key contributor to adaptive responses. Bioinformatic analyses have identified a single gene in all pathogenic and saprophytic *Leptospira* species that encodes a putative CdaA-like DAC. In *L. interrogans* strain Fiocruz L1-130, the putative DAC encoding gene is *lic10844*. Consistent with the properties of other CdaA-like DACs, the putative *Leptospira* DAC contains three N-terminal transmembrane (TM) domains and signature motifs (DGA and GXRHRXA) associated with the CdaA class of DACs (21). Henceforth, we refer to LIC10844 and the homologs encoded by other *Leptospira* spp. as CdaA. Here, we demonstrate that CdaA is antigenically conserved across diverse pathogenic *Leptospira* spp. and produced by all P1 subclade isolates tested during *in vitro* cultivation. Recombinant CdaA_88-273_ DAC activity was strictly dependent on the use of Co^2+^ or Mn^2+^ as cofactors. No DAC activity was detected when Mg^2+^, Zn^2+^, Cu^2+^, Ni^2+^, or Ca^2+^ were supplied as cofactors. The active form of CdaA was determined to be a homodimer. Single amino acid substitutions of specific residues within the ATP-coordination and putative active sites abolished or attenuated the enzymatic activity of recombinant CdaA_88-273_. Growth of *L. interrogans* in the presence of KCl, but not NaCl, resulted in a twofold upregulation of *cdaA* transcript levels. The results of this study suggest that *Leptospira* sp. produces an enzymatically active DAC that can produce c-di-AMP utilizing specific cofactors. This study is the first to demonstrate a link between CdaA activity and environmental adaptation in leptospires.

## RESULTS

### CdaA is conserved, expressed during cultivation, and localizes to the inner membrane

**Fig 1:**
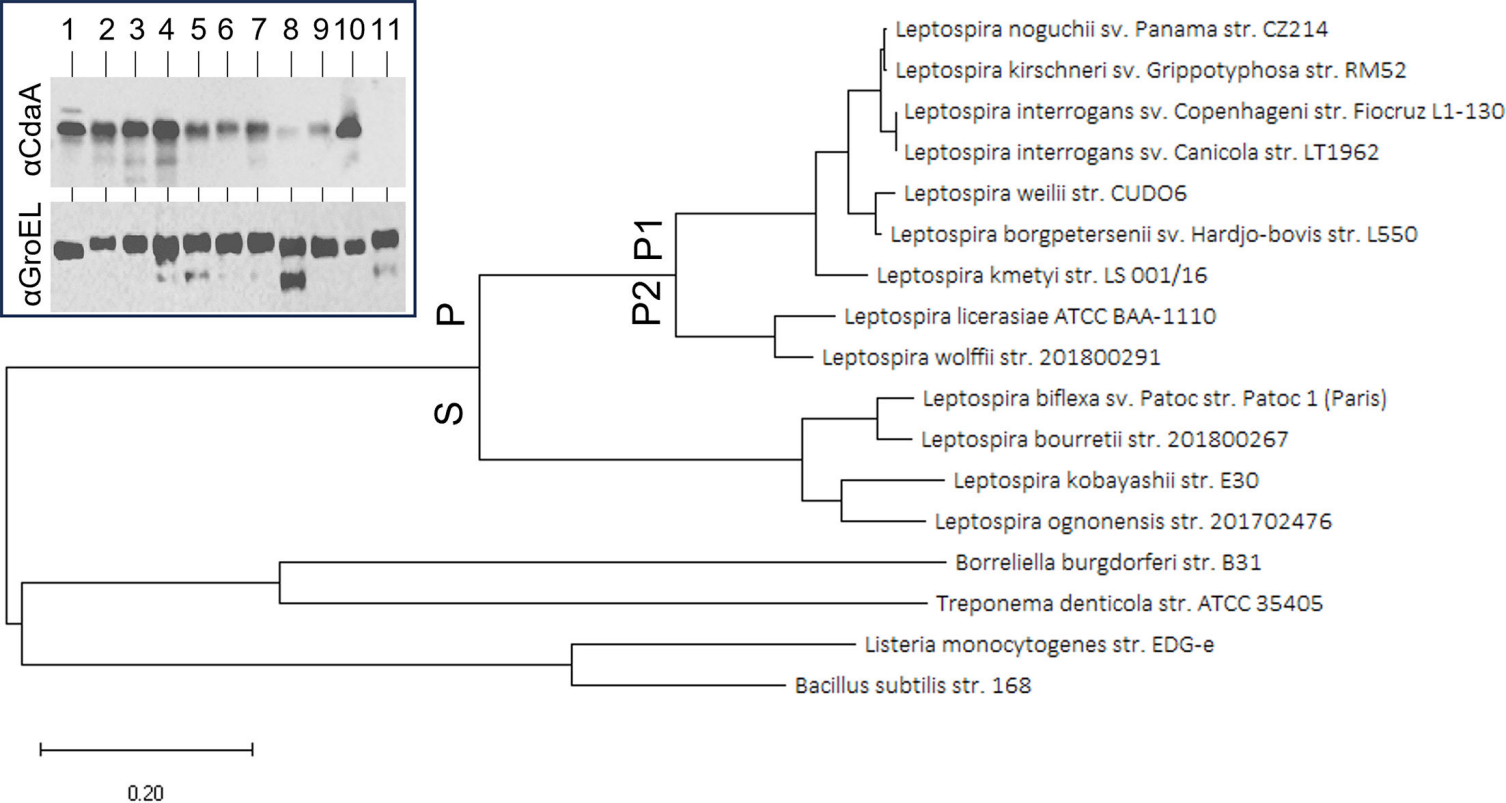
Phylogenetic and *in vitro* expression analyses of CdaA. Amino acid sequences of LIC10844 and homologues were retrieved from NCBI. The sequences were used to construct a neighbor-joining tree in MEGA11. A distance scale is included at the bottom left. The *Leptospira* pathogenic (P) and saprophytic (S) clades, as well as the pathogenic (P1) and intermediate (P2) subclades, are labeled. The insert is an immunoblot of cell lysates screened with anti-CdaA_88-273_ antiserum. Identical blots were screened with anti-GroEL as a loading control. The samples loaded in each lane are as follows: (1) *L. interrogans* str. Fiocruz L1-130, (2) *L. interrogans* str. Kito, (3) *L. interrogans* str. LC82-25, (4) *L. interrogans* str. L495, (5) *L. noguchii* str. Bonito, (6) *L. noguchii* str. Hook, (7) *L. noguchii* str. Cascata, (8) *L. kirschneri* str. RM52, (9) *L. borgpetersenii* str. M2, (10) *L. kmetyi* str. Bejo-Iso9T, and (11) *L. wolffii* str. Korat-H2T. All methods are detailed in the text. Note that the immunoblots were cropped for presentation purposes.

**Fig 2:**
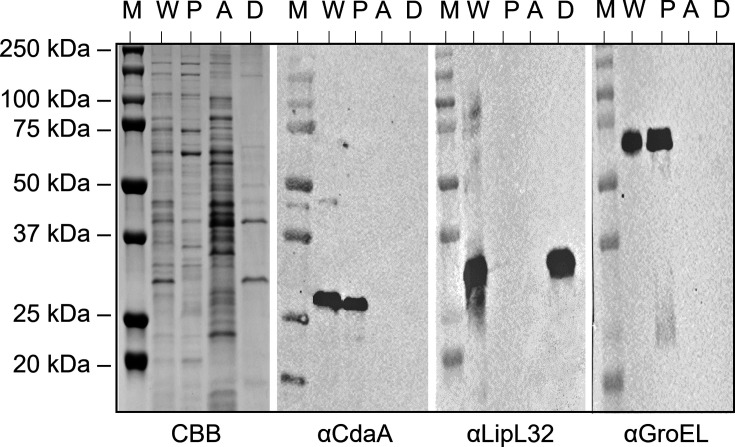
Localization of CdaA by Triton X-114 extraction and phase partitioning**.** *L. interrogans* str. Fiocruz L1-130 cells were extracted with Triton X-114 and phase-partitioned. A Coomassie Brilliant Blue (CBB) stain of the SDS-PAGE gel is shown on the left. A set of identical immunoblots was screened with anti-CdaA_88-273_, anti-LipL32, or anti-GroEL antiserum as indicated below each image. Molecular weight markers (M) are shown for each panel. Abbreviations are as follows: whole cell lysate (W), periplasmic cylinder (P), aqueous phase (A), and detergent-soluble phase (D). All methods are detailed in the text. Note that the immunoblots were cropped for presentation purposes.

A neighbor-joining phylogenetic tree delineated two distinct CdaA clades consistent with orthologous gene analyses that indicate separation of pathogenic (P1/P2) from saprophytic (S1) *Leptospira* species (Fig. 1) (25). CdaA percent amino acid identities respective to LIC10844 ranged from 88.7 to 99.0% among P1 strains, 72.0 to 74.6% among P2 strains, and 53.6 to 54.7% across saprophytic strains. The production of CdaA during *in vitro* cultivation was assessed by immunoblot analysis of P1/P2 strains (Table 1). Cell lysates were screened with anti-CdaA_88-273_ and anti-chaperonin GroEL antiserum (loading and detection control [26]). CdaA was produced by all strains, except for *L. wolffii* Korat-H2T (Fig. 1; insert). The basis for the lack of detectable CdaA in this strain remains to be determined. As expected, GroEL was detected in all strains. Triton X-114 extraction and phase partitioning coupled with immunoblot analyses demonstrated that CdaA localizes to the protoplasmic cylinder (Fig. 2). This observation and the presence of three predicted transmembrane domains in the N-terminal 87 amino acids of CdaA indicate that CdaA is an inner membrane associated with cytoplasmic catalytic domains. Immunoblots screened with anti-LipL32 (outer membrane) and anti-GroEL (cytoplasmic protein) served as localization controls, and both proteins were fractionated as expected (Fig. 2).

**TABLE 1:** Bacterial isolates utilized in this study

Isolate	Source
*Leptospira interrogans* serovar Copenhageni strain Fiocruz L1-130	Human, Brazil
*Leptospira interrogans* serovar Canicola strain Kito	Dog, Brazil
*Leptospira interrogans* serovar Pomona strain LC82-25	Human, USA
*Leptospira interrogans* serovar Manilae strain L495	Human, Philippines
*Leptospira noguchii* serovar Autumnalis strain Bonito	Human, Brazil
*Leptospira noguchii* serovar Australis strain Hook	Dog, Brazil
*Leptospira noguchii* serovar Bataviae strain Cascata	Human, Brazil
*Leptospira kirschneri* serovar Grippotyphosa strain RM52	Pig, USA
*Leptospira borgpetersenii* serogroup Ballum strain M2	Mouse, Puerto Rico
*Leptospira kmetyi* serovar Malaysia strain Bejo-Iso9T	Soil, Malaysia
*Leptospira wolffii* serovar Korat strain Korat-H2T	Human, Thailand

### CdaA is a functional diadenylate cyclase

**Fig 3:**
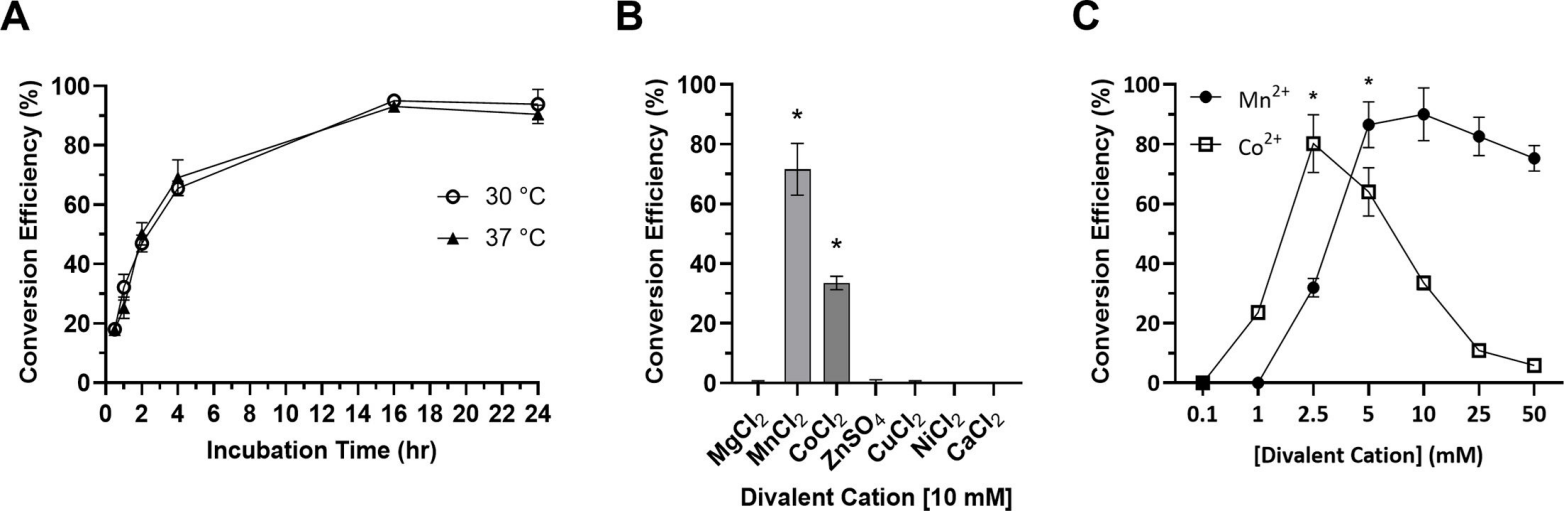
Analysis of the diadenylate cyclase activity of CdaA using coralyne fluorescence assays. The conversion of ATP to c-di-AMP by CdaA_88-273_ was assessed after 0.5, 1, 2, 4, 16, and 24 h at 30 and 37°C in 10 mM Mn^2+^ buffer (**A**). The activity of CdaA_88-273_ in the presence of different divalent cations (10 mM) was assessed after 16 h at 30°C (**B**). Significance (*P* < 0.05) values are shown in comparison to the buffer with ATP alone. In panel **C**, the DAC activity of CdaA_88-273_ was measured in the presence of increasing concentrations of MnCl_2_ and CoCl_2_ (16 h, 30°C). Asterisks represent the minimum required concentration of divalent cation to achieve maximal conversion efficiency (*P* < 0.05). Conversion of ATP to c-di-AMP was measured using coralyne fluorescence assays using a c-di-AMP standard curve. The results were confirmed by measuring conversion using RP-HPLC. All methods are detailed in the text. All assays were repeated at least three times.

To assess the DAC activity and divalent cation cofactor requirements of CdaA, a recombinant CdaA fragment spanning residues 88-273 (CdaA_88-273_) was produced with an N-terminal hexa-Histidine tag. Attempts to express full-length CdaA were unsuccessful most likely due to the N-terminal transmembrane domains. It has been demonstrated that removal of the transmembrane spanning domains of orthologous CdaA proteins does not affect the DAC activity (25, 27). The DAC activity was assessed over time in the presence of 10 mM MnCl_2_ at 30 and 37°C. No significant difference in the CdaA conversion efficiency was observed at 30°C versus 37°C (Fig. 3A). To determine CdaA divalent cofactor requirements, DAC assays were conducted in the presence of Mn^2+^, Mg^2+^, Co^2+^, Zn^2+^, Cu^2+^, Ni^2+^, and Ca^2+^. The DAC activity required specifically Mn^2+^ or Co^2+^ (Fig. 3B). The concentration of cobalt required for maximal conversion efficiency was half that of manganese (Fig. 3C), and CdaA was enzymatically active with as low as 1 mM cobalt versus a minimum of 2.5 mM manganese (Fig. 3C). These results highlight the potential utility of cobalt in environments where manganese availability may be limited.

### Identification of CdaA residues required for diadenylate cyclase activity

**Fig 4:**
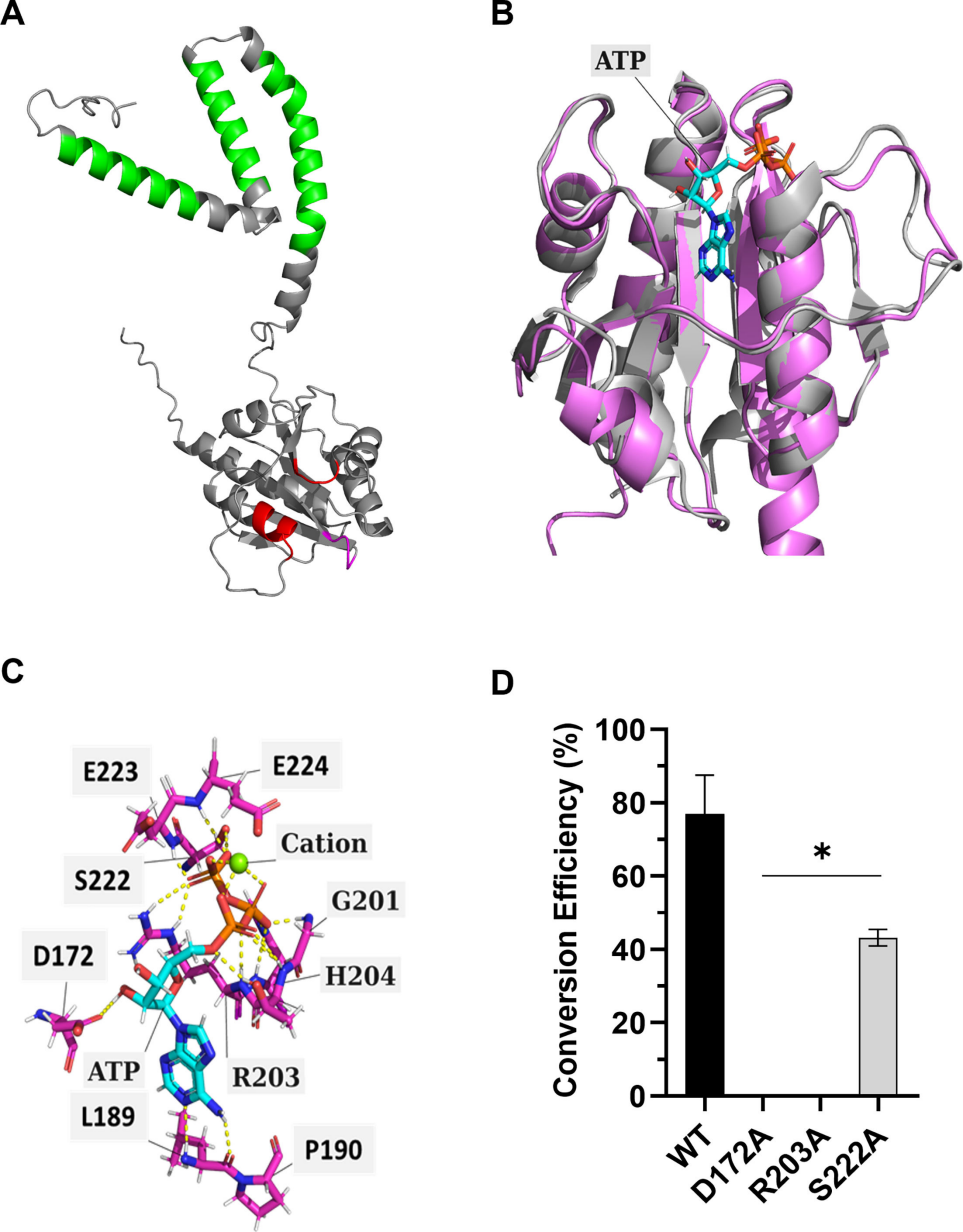
Site-directed mutagenesis of CdaA: identification of active site and ATP coordination residues. Panel **A** shows the AlphaFold 3 predicted structure of full-length CdaA with putative transmembrane domains in green, catalytic domains in red, and divalent cation coordination residues in magenta. In panel **B**, the predicted structure of CdaA (pink) is shown overlayed with the solved structure of *Listeria monocytogenes* CdaA (gray) in complex with ATP. In panel **C**, the residues of CdaA predicted to form polar contacts with either ATP or the cation co-factor are indicated. In panel **D**, the DAC activity of wild-type CdaA_88-273_ and CdaA_88-273_ proteins harboring site-directed mutations was measured using coralyne fluorescence assays. The results were confirmed by RP-HPLC area-under-curve analyses, as described in the text. Significance (*P < 0.05) was determined by one-way ANOVA relative to the sample containing WT CdaA_88-273_ protein. Note that the assays were repeated multiple times.

CdaA is predicted to contain three consecutive N-terminal transmembrane domains, the canonical “HDGA” and “GXRHRXA” catalytic domains, as well as “SEET” motifs, which coordinate the divalent cation (Fig. 4A). Although no crystal structure is available for any leptospiral CdaA protein, the structure of the orthologous *L. monocytogenes* CdaA in complex with ATP has been solved (27). Despite sharing just 35.8% amino acid identity, the AlphaFold three structural prediction of CdaA (LIC10844) and the solved crystal structure of the *L. monocytogenes* CdaA protein/ATP complex differ by only 0.529 Å (Fig. 4B). Polar contacts with ATP and/or the divalent cations in the *Listeria* CdaA-ATP complex were identified and extrapolated to the corresponding residues in *L. interrogans* CdaA (Fig. 4C). Based on these analyses, amino acid residues D_172_ (DGA active site), R_203_ (GXRHRXA active site), and S_222_ were targeted for substitution with Ala. S_222_ is predicted to coordinate the binding of ATP. The DAC activity of each mutated protein was measured after 0.5, 1, 2, 4, and 16 h (30°C) using two independent approaches: RP-HPLC and coralyne fluorescence assays. Substitution of D_172_ or R_203_ abolished DAC activity consistent with their location within the active site. The S_222_A mutated protein retained enzymatic activity, albeit with reduced conversion efficiency (47.5 ± 8.1%) (Fig. 4D). The decreased conversion efficiency presumably reflects perturbation of ATP binding.

### The active form of CdaA is a homodimer

**Fig 5:**
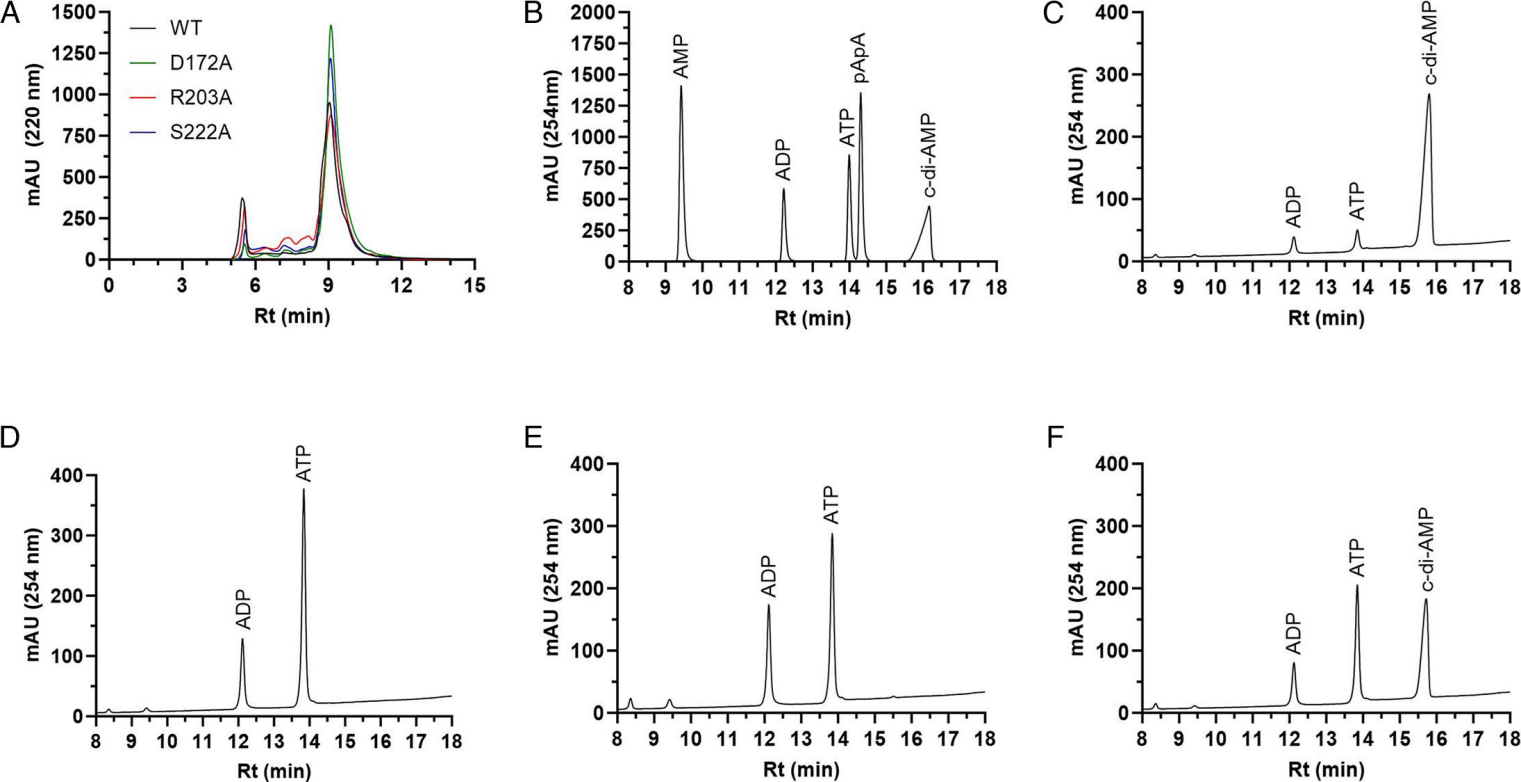
Analysis of the dimerization potential and diadenylate cyclase activity of CdaA and CdaA alanine substitution mutants. CdaA_88-273_ wild-type (WT) and alanine substitution mutants were assessed for their oligomerization state using size exclusion chromatography (**A**). The chromatograms for each protein are overlaid in panel **A** and differentiated by line color as indicated in the key. The proteins under peak 2 were collected, concentrated, and tested for DAC activity (**C–F**). Panel **B** shows the RP-HPLC chromatogram of relevant nucleotide standards. Panels **C–F** show RP-HPLC chromatograms of the DAC reactions conducted using CdaA_88-273_, CdaA_88-273_:D_172_A, CdaA_88-273_:R_203_A, and CdaA_88-273_:S_222_A, respectively. The identity of each nucleotide peak was determined based on the retention time (Rt) of the nucleotide standards shown in panel **A**. All methods are detailed in the text.

Recombinant CdaA_88-273_ and the D_172_A, R_203_A, and S_222_A amino acid substitution mutants were assessed by SEC, and the eluant under each peak was collected. The retention times of CdaA_88-273_ and the mutated proteins (peak 2; 9.1 min) were identical (Fig. 5A). An additional peak was observed at 5.5 min (discussed below). A standard curve was generated as described in the methods to derive a linear regression formula: Log(MW) = −0.3546 × (Rt(min)) + 4.843 (*R*^2^ = 0.9860). Based on this formula, the MW of the protein under peak two was estimated to be 41.3 kDa. This is consistent with a CdaA homodimer. Other CdaA-type DACs have also been demonstrated to form dimers. AlphaFold 3 prediction of the CdaA_88-273_ homodimer complex supported this conformation, with an iPTM score of 0.79. The MW of the protein that eluted under peak 1 (Rt = 5.5 min) was estimated to be 781.1 kDa. Note that this is outside of the linear range for size estimation. The protein under this peak is likely an aggregate artifact.

To determine whether homodimers of the wild-type and CdaA mutants are enzymatically active, the protein under peak two was collected, concentrated, and tested for DAC activity (Fig. 5A). The production of c-di-AMP was assessed using RP-HPLC. The elution profiles of the nucleotide controls (ATP and c-di-AMP) are presented in Fig. 5B. Dimeric CdaA_88-273_ converted ~83.5% of the input ATP to c-di-AMP after 16 h (Fig. 5C), demonstrating activity as a homodimer. Dimeric CdaA_88-273_:R_203_A and CdaA_88-273_:D_172_A were enzymatically inactive (Fig. 5D and E). CdaA_88-273_:S_222_A retained activity but at a reduced efficiency of 56.6%(Fig. 5F). The loss of enzymatic activity in CdaA_88-273_:R_203_A and CdaA_88-273_:D_172_A is consistent with their positions within the predicted DAC active site. The attenuated activity of CdaA_88-273_:S_222_A may reflect perturbation of the ATP-binding site.

### Transcription of cdaA is upregulated by potassium

**Fig 6:**
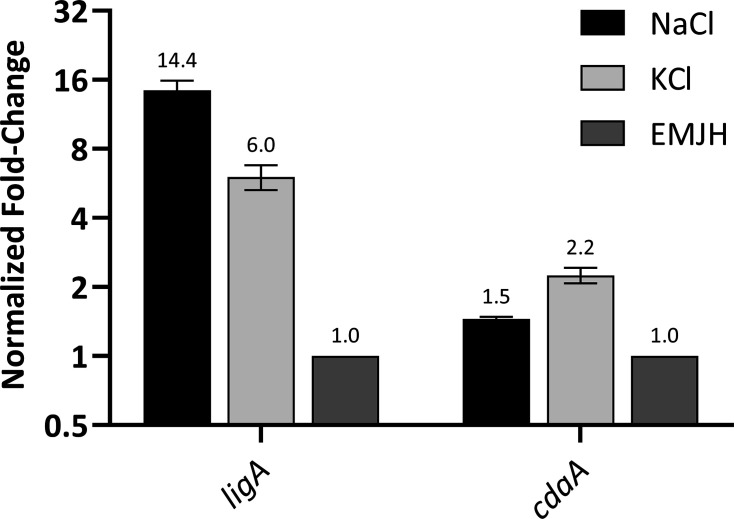
Potassium upregulates cdaA transcription. RT-qPCR was performed using c-DNA libraries generated using RNA extracted from *L. interrogans* strain Fiocruz L1-130 cultures that were propagated overnight with 50 mM NaCl, 50 mM KCl, or no added salt. RT-qPCR data of cdaA and ligA were normalized to rrs1, and fold-change relative to the EMJH group (no salt added) was calculated as described in the text. The average relative normalized fold-change values are provided above each bar. Fold change >2.0 versus EMJH was considered significant. Note that the assays were repeated multiple times.

To determine if sodium and potassium affect *cdaA* transcription, NaCl or KCl was added to early-log phase *L. interrogans* strain Fiocruz L1-130 cultures to a final concentration of 50 mM (in duplicate). The cultures were incubated overnight. Percent efficiencies of all primer sets were >90%, validating the use of the Livak method for subsequent relative fold-change calculations. The *ligA* gene was targeted as a known osmoregulated control (28), and *rrs1* was included for normalization. As expected, *cdaA*, *ligA*, and *rrs1* transcripts were not detected in controls lacking reverse transcriptase or DNA template (data not shown). Consistent with an earlier study that reported increased production of LigA when cells were exposed to 120 mM NaCl (28), we observed that *ligA* transcription was upregulated in cells grown in media supplemented with 50 mM NaCl (Fig. 6). In contrast, the *cdaA* expression was upregulated (2.2-fold) by KCl but not by NaCl (Fig. 6). These data suggest that potassium availability in nature may play a role in *cdaA* transcription and, by extension, influence c-di-AMP-mediated regulatory mechanisms.

## DISCUSSION

Our current understanding of the regulatory role of c-di-AMP in the pathogenesis and biology of *Leptospira*, as well as that of spirochetes in general, is limited. Bioinformatic analyses identified a single putative DAC in the genome of *Leptospira* species that we designate as CdaA. In this study, we characterized the properties of recombinant CdaA and assessed the influence of divalent cations and salts on enzymatic activity and transcription. Immunoblot analyses demonstrated that CdaA is expressed during cultivation by diverse pathogenic *Leptospira* spp. SEC (and AlphaFold 3 predictions) revealed that CdaA forms a homodimer, and subsequent DAC assays demonstrated that the homodimer is the biologically active form of the protein. Recombinant CdaA can utilize either Mn^2+^ or Co^2+^ for DAC activity, but not Mg^2+^ or other divalent cations. The CdaA protein of *L. monocytogenes* also functions as a homodimer and has the same divalent cofactor requirements (27, 29). The functionally related DisA (DNA integrity scanning protein A)-type DACs differ from CdaA-type DACs in that the active form is an octamer that requires Mg^2+^ for activity (30–32). DisA-type DACs exert their regulatory activity by binding to branched DNA structures (31). The effector mechanisms of CdaA-type DACs in spirochetes have not yet been determined.

The selective requirement of DACs for different divalent cations may correlate with amino acid sequence differences within their active sites (27). CdaA-type DACs have a conserved histidine that immediately precedes the “DGA” motif (29). DisA-type DACs, which utilize Mg^2+^ as a cofactor, do not have a histidine at the analogous position. The ability of CdaA to use cobalt as a cofactor may, in part, facilitate survival in nutrient- and divalent-cation-limited environments (27, 33). Vertebrates are an example of a nutrient-competitive climate. It has been postulated that pathogens that can utilize uncommon cations, such as cobalt, may have increased fitness in vertebrate hosts (33). Consistent with this, *L. interrogans* can invade and persist in nutrient-replete macrophages (34). Other pathogenic *Leptospira* species have not been evaluated for their ability to survive within macrophages. Manganese is crucial for bacterial defense against reactive oxygen species (ROS) (35, 36). Furthermore, the presence of manganese has been positively correlated with survival of leptospires in soil (37). The availability of divalent cations varies widely between the environmental and host phases of the leptospiral life cycle—even between different sites of the host—and could, therefore, modulate CdaA activity. Although leptospiral CdaA utilizes Mn^2+^ and Co^2+^ as cofactors, the relationship between c-di-AMP, ROS defense, and persistence in both the host and the environment has yet to be elucidated.

The *L. interrogans cdaA* gene is part of a 19-gene operon (38). The proteins encoded by the operon are involved in diverse functions including flagella structure, cell wall, and lipid biosynthesis. The first two genes of the operon encode the DapA and DapB proteins, both of which are involved in peptidoglycan synthesis and cross-linking. Following *dapB* are *cdaA* and *cdaR* (also referred to as YbbR [39]). In some gram-positive bacteria, CdaR negatively regulates DAC activity by binding to the N-terminal transmembrane spanning domains of CdaA (40). Notably, the *Leptospira* spp. *cdaA* operon lacks a GlmM homolog, which is typically present in operons that include *cdaA*. GlmM has been shown to regulate the DAC activity of some gram-positive CdaA proteins (39, 41). It is apparent that in *Leptospira* spp., the mechanisms of CdaA regulation, and by extension, c-di-AMP levels, are controlled using different mechanisms. Future work will seek to identify the mechanisms or factors that regulate CdaA production and thus c-di-AMP levels. Regulated control of c-di-AMP levels is essential, as high concentrations of c-di-AMP are toxic.

C-di-AMP has been shown to contribute to the transcriptional regulation of genes encoding proteins involved in ion uptake and transport by binding to riboswitches (42). In *B. subtilis*, increased K^+^ concentrations led to increased CdaA production (40), and elevated c-di-AMP levels in *Staphylococcus aureus* led to increased CpaA activity. CpaA is a K^+^ and Na^+^ cation/proton antiporter that binds c-di-AMP (43). In this study, we found that the addition of KCl, but not NaCl, to the cultivation media significantly increased *cdaA* transcription. Although all *Leptospira* spp. encode *cdaA*, the ability to regulate gene expression based on nutrient and osmotic conditions may contribute to the fitness of pathogenic strains when establishing infection in a host. It has been shown that exposure of *Leptospira* sp. to serum does not differentially alter *cdaA* expression *in vitro* but does cause a downregulation of the potassium-transporting ATPase, *kdpA* (44). Transcription of *kdpA* has been shown to be negatively regulated by c-di-AMP levels in *B. thuringiensis* via association with a riboswitch in the 5′-untranslated region of the *kdp* transcript (45). Riboswitches that bind c-di-GMP are known (17), but none have been identified that bind c-di-AMP in *Leptospira* species.

To assess the functional requirement for specific amino acids within the CdaA active sites (D^172^GA and “GXR^203^HRXA), Asp_172_ and Arg_203_ were individually substituted with Ala. Furthermore, Ser_222_ was substituted to examine the role of this site in the coordination of divalent cations and ATP. SEC demonstrated that these mutations do not interfere with homodimer formation. The D_172_A and R_203_A mutations within the putative active sites abolished DAC activity. Analysis of the crystal structure of *L. monocytogenes* CdaA complexed with ATP revealed that Ser_222_ directly interacts with the β- and γ-phosphates of ATP (27). Our data show that the loss of this ATP-coordination residue attenuates, but does not abolish, the conversion of ATP to c-di-AMP. Further mutational and structural analyses are required to elucidate the role of additional functional residues in dimerization and enzymatic activity.

In summary, pathogenic and saprophytic *Leptospira* encode a single, functional DAC (CdaA), which is the sole source of endogenous c-di-AMP. C-di-AMP has been shown in diverse bacteria to directly and indirectly regulate global metabolism, osmotic homeostasis, and stress responses (46), which are particularly relevant considering the range of hosts and environments in which leptospires can persist. In fact, c-di-AMP is the only cyclic dinucleotide demonstrated to be essential for viability in some bacteria (47). C-di-AMP can also influence the interactions of pathogens with the host immune system. The binding of c-di-AMP to STING receptors on eukaryotic cells stimulates a pro-inflammatory response (48) that affects the immune system’s ability to clear an infection (49). As a disease of inflammation, further research is needed to understand the role of this bacterial cyclic nucleotide and the host STING pathway in the onset of leptospirosis. Additionally, identification and characterization of c-di-AMP-specific binding targets and their effector mechanisms, as well as phosphodiesterases, will be essential to fully elucidate this complex and unexplored *Leptospira* regulatory system.

## MATERIALS AND METHODS

### Phylogenetic analyses

*Leptospira* spp. CdaA sequences were retrieved from NCBI, aligned using MEGA11 (50), and used to construct a neighbor-joining tree (100 bootstraps; Poisson amino acid substitution model; pairwise deletion of gaps). CdaA orthologues from *B. burgdorferi*, *T. denticola*, *Bacillus subtilis*, and *Listeria monocytogenes* served as outliers. Percent amino acid identity and similarity values were calculated using the “Ident and Sim” program (51).

### Bacterial cultivation

*Leptospira* isolates (Table 1) were cultivated (30°C) in EMJH medium supplemented with Probumin vaccine-grade bovine serum albumin (BSA; EMD Millipore) and 100 µg mL^−1^ of 5-fluorouracil, as previously described (52). Growth was monitored using wet mounts and dark-field microscopy. Cells were recovered by centrifugation and washed twice with Mg^2+^ supplemented phosphate-buffered saline (PBS; 137 mM NaCl, 5 mM MgCl_2_, 2.7 mM KCl, 2 mM KH_2_PO_4_, 1 mM Na_2_HPO_4_; pH 7.4).

### Generation and purification of recombinant proteins

All genes were synthesized fee-for-service (GenScript) and supplied in pET-45b(+). The DNA sequence encoding the N-terminal 87 amino acids was omitted from all constructs due to predicted transmembrane domains (DeepTMHMM) (53). *Escherichia coli* BL21 (DE3) cells (New England Biolabs) were transformed with the plasmids, and protein induction and protein purification were performed as previously described (54). In brief, protein expression was induced with isopropyl β-D-1-thiogalactopyranoside (IPTG; 1 mM), and cells were recovered by centrifugation and suspended in binding buffer (500 mM NaCl, 20 mM Na_2_HPO_4_, 20 mM imidazole; pH 7.4) supplemented with 1% protease inhibitor cocktail (PIC; Sigma-Aldrich) and 2.5 U mL^−1^ of Pierce Universal Nuclease (Thermo Scientific). Cells were lysed using an EmulsiFlex-C3 high-pressure homogenizer (three passes, 1,000–1,500 bar, 4°C). The soluble fraction was recovered by centrifugation, and the recombinant His-tagged proteins were purified by nickel affinity chromatography on an ÄKTA Pure 25 M FPLC (Cytiva) using 1 mL HisTrap FF columns (Cytiva) (14). Protein-containing eluates were pooled and dialyzed into PBS. Protein concentrations were determined by the bicinchoninic acid assay (Pierce), and purity was assessed by SDS-PAGE and Coomassie Brilliant Blue R-250 (Thermo Scientific) staining.

### SDS-PAGE and immunoblot analyses

*Leptospira* cell lysates were generated by suspension of washed cells (3.33 OD_600_ mL^−1^) in 1× SDS-PAGE buffer (10% glycerol, 2% SDS, 1% β-mercaptoethanol, 0.05% bromophenol blue, 125 mM Tris-HCl; pH 6.8). Cells were lysed by sonication and heated (10 min; 99°C). Recombinant proteins (100 ng μL^−1^) were prepared for SDS-PAGE by dilution in 1× SDS-PAGE buffer. Samples (5 μL) were heated (10 min; 99°C) and separated in Criterion TGX AnykDa gels (Bio-Rad) with Tris/glycine/SDS buffer (Bio-Rad). Proteins were visualized by staining with Coomassie Brilliant Blue R-250. Precision Plus Protein Dual Color Standards (Bio-Rad) served as the molecular weight (MW) markers. Stained proteins were imaged using the ChemiDoc Touch Imaging System (Bio-Rad) with the auto-optimal setting. Proteins were transferred to polyvinylidene difluoride (PVDF) membranes using the Trans-Blot Turbo System (Bio-Rad; high MW preset). Membranes were blocked (15 min) in EveryBlot blocking buffer (Bio-Rad). Primary antibodies (1:1,000 in blocking buffer) and goat anti-rat IgG horseradish peroxidase (HRP) secondary Ab (Novus Biologicals; 1:40,000 in blocking buffer) were incubated with blots for 1 h each. After each incubation with Ab, membranes were washed three times with PBST (0.2% Tween-20 in PBS). Immunoblots were incubated (5 min) with Clarity Western ECL Substrate (Bio-Rad) in the dark, and images were captured as above.

### Generation of hyperimmune serum

Anti-CdaA_88-273_ antiserum was generated in Sprague-Dawley rats (Charles River) as previously described (54). In brief, prime intraperitoneal vaccination consisted of 50 µg CdaA_88-273_ (1:1 Freund’s complete adjuvant; Sigma-Aldrich), followed by two booster doses of 25 µg protein (2 weeks apart; 1:1 in Freund’s incomplete adjuvant; Sigma-Aldrich). The rats were euthanized (CO_2_ inhalation, followed by cervical dislocation); blood was collected (cardiac puncture); and serum was harvested using VACUETTE Z serum Sep clot activator tubes (Greiner Bio-One). All animal studies were conducted in accordance with the Guide for the Care and Use of Laboratory Animals (Institute of Animal Research, National Research Council) under protocol AD10000387 approved by the Virginia Commonwealth University IACUC.

### Triton X-114 extraction and phase partitioning

Triton X-114 extraction and phase partitioning were performed as previously described (55), with some modifications. Approximately 10^10^ mid-log phase *L. interrogans* strain Fiocruz L1-130 cells were recovered by centrifugation and washed twice with wash buffer (5 mM MgCl_2_ in PBS, pH 7.4). Cells were suspended at 3.0 OD_600_ mL^−1^ in wash buffer, centrifuged, suspended in 1 mL of extraction buffer (1% Triton X-114, 1% PIC, 150 mM NaCl, 10 mM Tris-HCl [pH 7.5], 1 mM EDTA), and incubated on ice for 2 h. The samples were centrifuged to separate the soluble (S) and insoluble protoplasmic cylinder (*P*) fractions. The *P* fraction was washed twice with 1 mL of extraction buffer, and then stored dry at −20°C. The S fraction was treated with 20 mM CaCl_2_, incubated (37°C, 15 min), then centrifuged to separate the aqueous phase (A) from the detergent-soluble phase (D). The A and D phases were washed twice with 500 µL extraction buffer and wash buffer, respectively. Proteins in the A and D phases were precipitated with 10 volumes of ice-cold acetone (rocking on ice; 30 min) and recovered by centrifugation. After evaporation of the acetone, samples were resuspended in PBS and analyzed by SDS-PAGE as above.

### Diadenylate cyclase (DAC) assays

DAC assays were performed as described (56), with some modifications. In brief, 5 μM recombinant protein and 150 μM ATP (New England Biolabs) were incubated at 30 and 37°C for 0.5, 1, 2, 4, 16, and 24 h in DAC buffer (50 mM Tris HCl, 50 mM NaCl, 0.5 mM EDTA supplemented with 10 mM MgCl_2_, MnCl_2_, CoCl_2_, ZnSO_4_, CuCl_2_, NiCl_2_, or CaCl_2_; pH 7.5). The samples were heated at 95°C for 5 min, centrifuged (15,200 × *g*; 2 min), and filtered (0.22 μm PVDF membrane; EMD Millipore) into amber fixed insert vials (Agilent) for immediate analysis.

### Reverse-phase high-performance liquid chromatography (RP-HPLC)

RP-HPLC was performed as previously described (56) with some modifications. DAC reactions and reference standards (150 μM ATP and c-di-AMP; Millipore Sigma) were separated in a Supelcosil LC-18-T (3 μm) column (Millipore Sigma) on a 1260 Infinity II HPLC System (Agilent). Samples were injected (20 μL) into the equilibrated column. A linear gradient of buffers A (100 mM KH_2_PO_4_, 4 mM tetrabutylammonium hydrogen sulfate; pH 5.9) and B (100% methanol) was generated, starting with 100% Buffer A, increasing to 50% Buffer B over 20 min at a flow rate of 0.7 mL min^−1^. Retention times (Rt; min) were measured at 254 nm. The conversion efficiency for each sample was calculated from area-under-the-curve analyses relative to the known amount of the c-di-AMP standard.

### Fluorescence-based detection of c-di-AMP

The production of c-di-AMP in DAC reactions was measured using a fluorescence-based coralyne assay (57). In brief, DAC assays were run as above. Master mixes (100 μL) comprised 65% DAC reaction, 25% quenching buffer (1 M KBr, 150 mM NaCl, 25 mM Tris; pH 7.2), and 10% coralyne solution (150 mM NaCl, 25 mM Tris, 100 μM coralyne chloride); pH 7.2 (Sigma-Aldrich) was prepared, and 30 μL aliquots were transferred to black 384-well plates (Corning) in triplicate. The samples were mixed using an orbital shaker, and then read on a BioTek Synergy H1 microplate reader at λ_ex_ = 420 nm (27 nm bandwidth) and λ_em_ = 540 (25 nm bandwidth). Concentrations of c-di-AMP in each DAC reaction were determined relative to the fluorescence of the c-di-AMP standard.

### Structural predictions of CdaA

The structure of CdaA was predicted with AlphaFold 3 (58) using the ChimeraX application (59) and superimposed onto the crystal structure of *L. monocytogenes* CdaA in complex with ATP and divalent cation (4RV7) (27). Root mean square deviation (RMSD) between the two structures was calculated using the PyMOL Molecular Graphics System, Version 4.6.0 (Schrödinger, LLC). Residues predicted to form polar contacts with ATP and/or the divalent cation were identified using PyMOL.

### Size exclusion chromatography (SEC)

Size exclusion chromatography (SEC) was performed as previously described (15) using a TSKgel G4000SW_XL_ column (Tosoh Bioscience) and a 1260 Infinity II HPLC System. PBS served as the mobile phase. A standard curve was generated using 15–600 kDa standard protein mix (Millipore Sigma). The standards were prepared according to the manufacturer’s instructions, filtered through a 0.22 µm PVDF membrane into an amber fixed insert vial, and injected (20 μL) into the column. They were then eluted with PBS (1.0 mL min^−1^). Retention times (Rt; min) were plotted against log(MW) to fit a linear regression model. To assess the oligomeric state of CdaA proteins, 50 µg of protein (in PBS) was filtered as above, injected (25 μL) into the column, and eluted with PBS (1.0 mL min^−1^). Protein under each peak (254 nm) was collected and concentrated using Amicon Ultracel 10,000 NWML 0.5 mL centrifugal filters (Millipore Sigma) for subsequent DAC assays.

### Reverse transcription quantitative polymerase chain reaction (RT-qPCR)

Low-passage *L. interrogans* strain Fiocruz L1-130 cells were grown in EMJH medium to early-log phase, as described above. Two cultures per group were then supplemented with 50 mM NaCl, 50 mM KCl, or EMJH alone and incubated overnight at 30°C. Cells were collected by centrifugation (10,000 × *g*, 10 min, 4°C), and the supernatants were decanted. RNA was then extracted using TRIzol reagent according to the manufacturer’s instructions (Thermo Fisher Scientific). RNA concentrations were determined using a Qubit RNA Broad-Range Assay Kit (Thermo Fisher Scientific), and RNA quality and integrity were assessed using the Qubit RNA IQ Assay Kit (Thermo Fisher Scientific). Samples were diluted with nuclease-free water (200 ng µL^−1^), and residual genomic DNA (gDNA) was removed using the TURBO DNA-Free Kit (Thermo Fisher Scientific) according to the manufacturer’s instructions. Complementary DNA (cDNA) libraries were constructed from 0.5 to 1 µg of RNA using iScript Reverse Transcription (RT) Supermix for RT-qPCR (Bio-Rad) according to the manufacturer’s instructions. A no-RT reaction was generated for each RNA sample using the iScript No-RT Supermix to control for potential carryover gDNA contamination.

RT-qPCR primers were designed to amplify *cdaA*, *ligA* (LIC10465), and *rrs1* (LIC11010) (Table 2). The *ligA* gene was targeted as a positive control, as its transcription is known to be regulated by osmolarity (28). The amplification efficiency of each primer set was determined using 10-fold serial dilutions (10^6^ to 10^0^ genome equivalents) of *L. interrogans* strain Fiocruz L1-130 gDNA purified with a DNeasy Blood and Tissue Kit (Qiagen) according to the manufacturer’s instructions. The concentration of gDNA was determined using a Qubit 1× dsDNA Broad-Range Assay Kit (Thermo Fisher Scientific), and genome equivalents per µL were calculated based on the known genome size of strain Fiocruz L1-130 (4,627,366 bp). A no-template control was also included for each primer set. RT-qPCR reactions were performed in triplicate using hard-shell 384-well clear/white PCR plates (Bio-Rad) in a CFX Opus 384 Real-Time PCR System (Bio-Rad) with SYBR Select Master Mix (Thermo Fisher Scientific) according to the manufacturer’s instructions. The Cq values and melt curves for all reactions, as well as the standard curves and percent efficiencies for both primer sets, were determined using Bio-Rad CFX Maestro software (default settings). The results were then exported to Excel for subsequent analysis. All *cdaA* and *ligA* readouts were normalized to *rrs1*, and fold change relative to the untreated culture was determined using the Livak method (2^−ΔΔCq^). A relative normalized fold change greater than 2 was considered significant. Two independent experiments were performed, each in duplicate.

**TABLE 2:** RT-qPCR oligonucleotide primers

Primer name	Sequence (5′–3′)
*cdaA* FWD	CTGTGCAGTTAGACGCGATCATCTC
*cdaA* REV	CCAAGTGCGGCTCTGTGTCTG
*ligA* FWD	CTCTCCAACTCGCGCTTCGATTG
*ligA* REV	CGAAGAAGACTTCCAGGTGACTTGCTC
*rrs1* FWD	ACTGGATGGTCCCGAGAGATCATAAG
*rrs1* REV	CCTCACCAACTAGCTAATCGGACG

### Statistical analyses

Ordinary one-way analysis of variance (ANOVA), followed by Dunnett’s multiple-comparison test, and two-way ANOVA fit with a full model were performed in GraphPad Prism 10.3.1 for Windows (GraphPad Software; https://www.graphpad.com/).
